# High-Yield Production of Lignin-Derived Functional Carbon Nanosheet for Dye Adsorption

**DOI:** 10.3390/polym12040797

**Published:** 2020-04-02

**Authors:** Fenggui Chen, Xi Hu, Xiaohan Tu, Linfei Chen, Xi Liu, Linli Tan, Yulin Mao, Jianwei Shi, Xiaoxu Teng, Shuhua He, Zonghui Qin, Jianhua Xu, Jian Wu

**Affiliations:** 1 Chongqing Key Laboratory of Inorganic Special Functional Materials, School of Chemistry and Chemical Engineering, Yangtze Normal University, Chongqing 408100, China; fgchen@yznu.cn (F.C.); 18290323757@163.com (X.H.); 17623843975@163.com (X.T.); c1834524995@163.com (L.C.); l17623663618@163.com (X.L.); tan13896294019@163.com (L.T.); m15736519396@163.com (Y.M.); thshuhua@163.com (S.H.); qinzonghui654321@163.com (Z.Q.); xujianhua64@163.com (J.X.); 2 Key Laboratory of Magnetic Materials and Devices, Ningbo Institute of Materials Technology and Engineering, Chinese Academy of Sciences, Ningbo 315201, China

**Keywords:** lignin, carbon nanosheet, catalyst, freeze-drying, carbonization, high yield

## Abstract

In this article, we report the preparation of lignin-derived carbon nanosheet (L-CNS) by direct thermal treatment of lignin without activation operation and the functions of the L-CNS as an adsorbent for rhodamine dye. The L-CNSs are fabricated by freeze-drying (FD) methods of lignin followed by high-temperature carbonization. It is found that lower frozen temperature in FD or lower concentration of lignin aqueous solution renders L-CNSs’ more porous morphology and higher specific surface area (SSA), allowing a promising application of the L-CNSs as an efficient adsorbent for organic pollutants. In particular, the alkaline hydroxide catalyst helps to increase the SSA of carbon products, leading to a further improved adsorption capacity. On the other hand, p-toluenesulfonic acid (TsOH) catalyzed pyrolysis, which dramatically increased the L-CNS product yield, and provided a high-yield approach for the production of pollutant absorbent.

## 1. Introduction

Owing to the wastewater discharges into rivers, severe environmental problems were caused by some industries; thus, the natural streams have spurred great attention to developing efficient adsorbents in order to remove pollutants in water purification [1,2,3]. Known that, rhodamine dye, a highly carcinogenic substance widely used as a colorant in the manufacturing of textiles and food products, could lead to subcutaneous tissue bore sarcoma [4,5]. As a result, the development of the adsorbents, which are usually produced from coal or wood, with high adsorption capacity for pollutant control has drawn extensive attention [6,7,8]. Increasingly expected, agricultural, renewable and low-cost materials or even organic wastes as hemicelluloses, cellulose, coconuts husk, and lignin should be of priority [9,10,11,12]. 

As known, lignin is an abundant natural macromolecular material, forming the chief part of woody plant tissues with cellulose, whose usual disposal as a waste product in the paper industry poses a major problem [13,14]. Besides, lignin possesses rich aromatic, phenolic, and aliphatic hydroxyl groups in its three-dimensional-branched chemical structure [15]. Since this special structure is obtained at a low cost, increased attention has recently been devoted to the development of value-added applications for lignin [16,17]. Hence, some lignin derivatives were employed in the application of water treatment, for instance, lignin itself exhibits high adsorption capacities for heavy metal ions owing to its abundant phenolic groups and can be a good adsorbent for pollutants over wide concentration ranges [18,19,20]. However, lignin usually disperses in aqueous solution, causing secondary pollution. Thus, a cross-linked lignin aerogel, which acted as an efficient adsorbent for organic dyes and heavy metal ions, was fabricated by freeze-drying of lignin followed by low-temperature annealing in our previous work [21]. However, most of the lignin-derived adsorbents exhibit low adsorption capacity and are prepared by complex methods. Thus, an economical and sustainable method is highly required to fabricate lignin-based materials for pollutant removal. 

It is also noteworthy that lignin, accounting for nearly 30% of the organic carbon on earth, can be used as an excellent precursor to prepare carbon-based materials [22,23,24], like activated carbons [20]. With large surface area, it can act as an excellent adsorbent to adsorb metal ions or organic molecules [25]. Up to now, active carbons with high SSA are typically prepared via activation to produce high micropore volumes, wide micropore sizes, and the desired surface functional groups [26,27]. As known, freeze-drying (FD) was reported of a very light aerogel formation with the extremely large specific surface area [28,29,30]. Besides, since alkaline lignin can be well dispersed in water and has a three-dimensional-branched structure with numerous aromatic units, it can also form a porous morphology upon FD of its aqueous solution, resulting in the lignin-based aerogel [21]. 

Therefore, a novel, simple, and efficient method was developed to fabricate the porous lignin-derived carbon nanosheet (L-CNS), which can be used as an excellent adsorbent for pollutant as rhodamine dye, via freeze-drying (FD) and subsequently, high-temperature carbonization without a separate activation operation. On the other hand, inspired by the chemical activation using alkaline hydroxide [31], activation also can be operated during the carbonization process to further increase their SSA, to verify the hypothesis. The improved SSA by simple activation operation in the structure under the high-temperature treatment will further enhance adsorption of water pollutants. Moreover, an ultrathin carbon nanofiber (CNF) aerogel from the wood-based nanofibrillated cellulose aerogels was successfully synthesized via a catalytic pyrolysis process using p-toluenesulfonic acid (TsOH) as catalyst, which dramatically enhances the carbon residual and well maintains the nanofibrous morphology [8]. Therefore, by introduction of TsOH into the lignin aerogel precursor, the product yield of our lignin-derived activated carbon aerogel with desired surface chemistry and high specific surface area is expected to be improved, providing a promising approach for high-yield production of low-cost adsorbent for pollutant in water treatment. 

## 2. Materials and Methods 

### 2.1. Materials

Water-soluble alkali lignin was purchased from TCI America and used as received. Rhodamine B (RB) and other chemicals were purchased from Sigma-Aldrich Chemicals Incorporation (Louis, MO, USA) and used without further purification. All solutions were prepared by the use of deionized (DI) water.

### 2.2. Preparation of Lignin-Derived Carbon Adsorbents

Water-soluble lignin aqueous solutions with concentrations of 50, 20, 10, and 5 mg/mL were prepared by dissolving lignin in DI-water and subsequent ultrasonic bath for 1 h. These four solutions were allowed to freeze at either −20 °C or −196 °C (Figure S1), then freeze dried in vacuum for 3 days, fabricating lignin aerogels with different densities. 

Afterwards, the obtained aerogels were firstly heated to 250 °C at a heating rate of 5 °C/min, followed by keeping them at 250 °C for 0.5 h. Then, the samples were heated to 1000 °C at 3 °C/min and held at this temperature for 1 h. After that, the lignin-derived carbon aerogels were obtained after cooling to room temperature. All carbonizations were carried out in a tube furnace (Hefei Kejing, OTF-1200X, Hefei, China) and in the Ar_2_ atmosphere condition.

### 2.3. Characterization

The morphologies of the obtained lignin-derived carbons were examined by the use of a field emission scanning electron microscope (FESEM, JEOL JSM 6340F, Tokyo, Japan) after coating with gold. N_2_ adsorption/desorption was performed by Brunauer–Emmett–Teller (BET) measurement with a Tristar-3000 surface area analyzer (Micromeritics Instrument Corp., Norcross, GA, USA), and the specific areas were calculated by the Barrett−Joyner−Halenda (BJH) method. The lignin-derived carbon adsorbents were placed in 10 mL of RB aqueous solution with concentrations of 10 mg/L, and UV–Vis spectrophotometer (Shimadzu UV–Vis 2501PC, Tokyo, Japan) was used to measure the UV–Vis absorbance spectra of RB solution at wavelengths ranged from 200 to 700 nm. RB adsorption amounts on the adsorbents were calculated using qt = (C0 − Ct)V/m, where qt (mg/g) is the adsorption capacity; C0 is the initial concentration of RB (10 mg/L); Ct (mg/L) is the RB concentration after adsorption; V (mL) is the solution volume; and m (g) is the adsorbent dosage.

## 3. Results and Discussion

### 3.1. Effects of FD and Freezing Temperature

**Figure 1:**
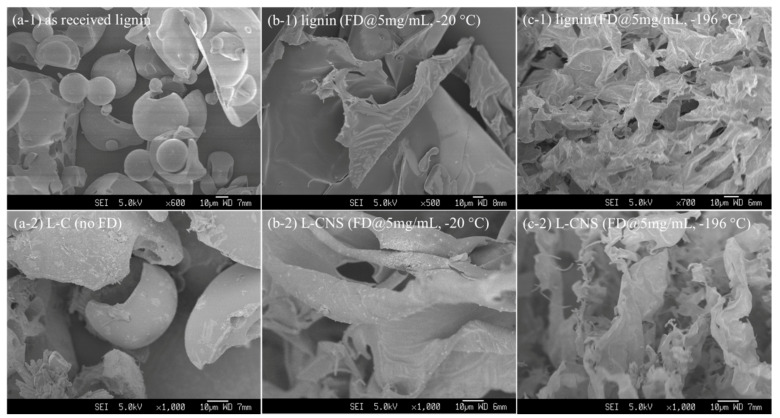
SEM images of the as-received alkali lignin before (**a-1**) and after carbonization (**a-2**), freeze-dried lignin frozen at −20 °C before (**b-1**) and after carbonization (**b-2**), and freeze-dried lignin frozen at −196 °C before (**c-1**) and after carbonization (**c-2**). The concentration of the lignin aqueous solutions used for FD was 5 mg/mL. The carbonization temperature was 1000 °C.

In order to obtain an adsorbent with a large specific surface area, lignin aqueous solution was freeze-dried before carbonization. To investigate the effect of FD on lignin and lignin-derived carbon nanosheets (L-CNSs), samples were prepared with and without FD. Freeze-dried samples were prepared at two different freezing temperatures: −20 °C and −196 °C. All the prepared samples were then carbonized at 1000 °C, and then compared with their lignin precursors in terms of morphology and specific surface area. Figure 1 shows the morphologies of the lignin precursors and their corresponding L-CNSs. As can be seen, lignin without FD (a-1) and its corresponding lignin-derived carbon (L-C, a-2) consist of particles; however, lignin precursor prepared by FD of 5 mg/mL lignin aqueous solution frozen at −20 °C forms a continuous porous sheet morphology (b-1) which is maintained almost entirely after carbonization (b-2). Figure 1c-1 shows that porosity increases as the freezing temperature drops from −20 °C to −196 °C. Table 1 indicates that FD both increases the specific surface area and pore volume and also decreases the average pore volume of L-CNSs by two orders of magnitude. The correspond N_2_-adsorption isotherms are provided in Figure S2. The above results demonstrate that FD is an effective route to produce lignin-based carbon aerogels. 

The reason for the rise in porosity is that ice crystals formed at super-cooling temperatures displace lignin to certain regions [32]. After FD, ice crystals sublimate and leave a porous lignin matrix behind. Since freezing temperature can affect nucleation and growth of ice crystals, it can determine the size and shape of pores, pore size distribution, and pore connectivity of the porous matrix, i.e., lignin. Nucleation creates a stable interface that provides a structural template for water molecules to deposit on. Nucleation can be either primary or secondary. Since there was no pre-existing crystal in our solutions at the time of freezing, only primary nucleation was present. Primary nucleation itself can be either heterogeneous, caused by foreign particles, e.g., lignin, or homogenous. Homogenous nucleation is a process through which a sufficient number of water molecules, typically 70–210, gain spontaneous order as a result of relatively long-ranged hydrogen bonds and intrinsic fluctuations in the medium. It requires a larger super cooling driving force, i.e., at temperatures below −31.15 °C. Crystal growth, which can also be affected by freezing temperature, consists of the diffusion of water molecules from the solution to the interface, and counter-diffusion of the solute, i.e., lignin, away from the growing crystals.

**Figure 2:**
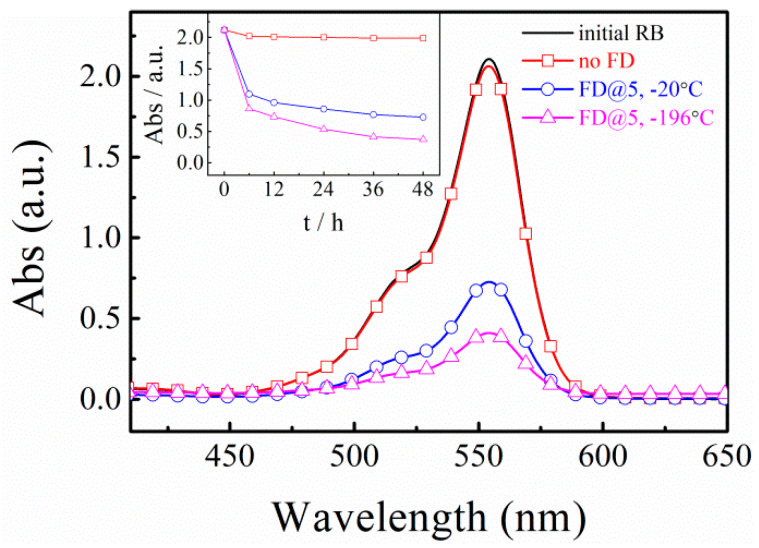
UV–Vis absorption spectra of RB solutions (initial RB conc. = 10 mg/L) before and after 48 h adsorption by 2 mg of L-CNSs. The samples were prepared by carbonization without prior FD (No FD) and with prior FD of solutions frozen at −20 °C and −196 °C (FD@-20 and FD@-196). The concentration of the freeze-dried solutions was 5 mg/mL. Inset: corresponding UV–Vis absorptions at ~554 nm as a function of adsorption time.

Since the concentration of lignin was kept constant in our comparisons, it can be assumed that the main difference between low and high freezing temperatures is the homogenous nucleation. At −196 °C, in addition to heterogeneous nucleation caused by lignin, homogenous nucleation contributes to the number of nuclei as well; hence, water molecules are divided among more nuclei, resulting in the mean size of crystals to diminish. Besides, since the freezing rate is high, water has less time for displacement, and consequently, these smaller crystals are more uniformly distributed throughout the lignin matrix. As a result, at −196 °C, the higher freezing rate results in a higher number of smaller crystals that are more uniformly distributed, yielding a smaller pore volume and a more porous structure in lignin after FD. On the contrary, freezing at −20 °C yields a fewer number of larger crystals that are less uniformly distributed because of more water dislocation; therefore, larger pore volume and less porosity can be observed in Figure 1c-1,c-2 compared to Figure 1b-1,b-2, respectively. 

RB is an organic aromatic chemical widely used as an excellent tracer dye in the fluorescence-related biotechnology [33,34]. Here, we used RB to study the adsorption properties of L-CNSs. Figure 2 shows the UV–Vis absorption spectra of 10 mL RB aqueous solution with an initial concentration of 10 mg/L after 48 h adsorption by 2 mg of L-CNSs. Clearly, the L-CNSs prepared without FD does not affect the spectra at 554 nm noticeably. By contrast, L-CNSs produced after FD show much lower UV–Vis absorption, indicating a lower residual concentration of RB and a higher RB adsorption capacity. Figure 2 also shows that decreasing the freezing temperature from −20 °C to −196 °C produces L-CNS with higher RB adsorption capacities. Besides, there are only decreases of the UV–Vis absorption intensity without shape change of the UV–Vis absorption spectra and no shift of absorption wavelength. Figure 2 inset shows the UV–Vis absorption of RB at ~554 nm as a function of contact time between RB solutions and 2 mg of different L-CNSs. As can be seen, the L-C produced without FD does not affect the adsorption even after 48 h. However, upon FD, the adsorption plummets for both L-CNSs prepared via FD, most significantly in the first 6 h. After the initial 6 h, the reduction in absorption slows down and reaches a plateau in the final hours of the measurements. The UV–Vis absorption results agree well with the specific surface area measurements. Since, the difference in the specific surface area values of L-CNSs prepared with and without FD is as large as two orders of magnitude, the difference in their UV–Vis spectra is significant as well (Figure 2). However, because reducing the freezing temperature from −20 °C to −196 °C increases the specific surface area only by twofold, the difference between the UV–Vis absorptions is less significant. Since freeze-dried L-CNSs at −196 °C showed the largest specific surface area and the highest adsorption capacity, the effects of other factors on properties of L-CNSD adsorbents were investigated using the L-CNSs prepared from solutions frozen at −196 °C.

### 3.2. Concentration Effects of Lignin Aqueous Solution

**Figure 3:**
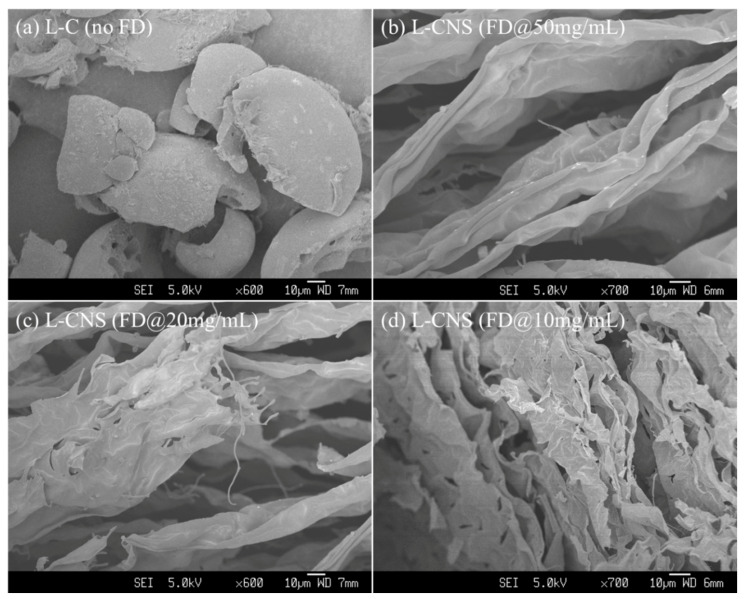
SEM images of the L-CNSs prepared by FD of lignin aqueous solutions, frozen at −196 °C, with varying concentrations, (**a**) as-received, (**b**) 50, (**c**) 20, and (**d**) 10 mg/mL, followed by carbonization at 1000 °C.

Lignin concentration in the aqueous solution that is used for FD may influence the morphology and properties of lignin aerogels and their corresponding L-CNSs when the lignin concentration is above the critical micelle concentration of lignin in water [28]. Hence, lignin aqueous solutions with concentrations of 50, 20, 10, and 5 mg/mL, frozen at −196 °C, were freeze-dried and carbonized at 1000 °C. Figure 3 shows SEM images of the L-CNSs with lignin precursor concentrations of 50 (a), 20 (b), 10 (c), and 5 mg/mL (d). As can be seen, the L-CNSs prepared by FD of high-concentration lignin aqueous solutions have a much thicker and larger sheet structure compared to the ones with lower concentrations. Besides, upon decreasing lignin concentration from 50 to 5 mg/mL, the appeared large sheets turn into the small sheet structures. Table 2 lists the pore volume and specific surface area values of the L-CNSs. The correspond N_2_-adsorption isotherms are provided in Figure S3. The BET analysis shows that as lignin concentration is decreased successively from 50 to 5, specific surface area value rises steadily. However, as the lignin concentration is halved from 20 to 10 mg/mL, a significant rise in specific surface area value can be seen. Pore volume, also, increases steadily by increasing lignin concentration, which agrees with SEM images in Figure 3. 

Based on the above studies, lignin concentration in the lignin aqueous solution used for FD greatly influences the morphology of the L-CNSs, which consequently can affect the dye adsorption capacity of these materials. Hence, we conducted RB adsorption measurements for these L-CNSs. Figure S4 shows the UV–Vis absorption spectra of RB solutions after 48 h of contact time with L-CNSs. The adsorption capacities of lignin-derived carbons for RB solution with a concentration of 10 mg/L under as different lignin concentrations for FD are listed in Table 2. The graph shows that the L-CNS prepared by FD with 50 mg/mL lignin concentration has a slightly better adsorption capability than the L-CNS prepared without FD, which adsorbs almost no RB. This improvement in RB adsorption can still be enhanced, albeit less significantly, by reducing lignin concentration to 20 mg/mL. Interestingly, reducing the lignin concentration to 10 mg/mL brings about a profound positive change in the RB adsorption capacity of the corresponding L-CNSs. The best result was observed for lignin concentration of 5 mg/mL. That is, with the decrease of lignin concentration, the L-CNS possesses a remarkable increase of the RB adsorption capacity. It is highly consistent with the result of our previous work that lignin-derived carbon nanofiber, prepared with lignin concentration of 1 mg/mL, has the RB adsorption capacity up to 170.4 mg/g. Moreover, RB adsorption capacities of these L-CNSs, listed in Table 2, reveal a negative correlation between the concentration of lignin in the freeze-dried solutions and the adsorption capacity of obtained L-CNSs, which is consistent with the results presented in Figure 3 and Table 2. We have established a positive correlation between specific surface area and lignin concentration. 

Besides, the product yields of L-CNSs are also significantly affected by lignin concentration for FD (Table 2), as lignin concentration is decreased successively from 50 to 5 mg/mL, the product yield decreases steadily. The L-CNS prepared by 50 mg/mL lignin solution possesses the highest yield, up to 41.9%, which increases by 166% compared to the one from 5 mg/mL. It is probably because the more dilute lignin aerogel allows the easier emission of the CO_2_ during the annealing process.

### 3.3. Catalysts for Lignin Carbonization

We demonstrated that RB adsorption capacities of L-CNSs reduce as specific surface area drops. In order to further enhance the adsorption capacity, the larger SSA is required. As known, activated carbon can be synthesized by carbonization, and the following chemical activation using alkali materials like NaOH as the activating agent. Therefore, NaOH was also employed and introduced into the lignin to activate the lignin-derived carbon to obtain higher SSA. Differently, lignin was doped into lignin precursors in the freeze-drying process, and the carbons were produced after carbonization operation, giving rise to high specific surface area and consequently, significant RB adsorption due to Van der Waals interactions. As can be seem in Table 3 and Figure S6, showing the UV–Vis absorption spectra of 10 mL RB aqueous solution with an initial concentration of 10 mg/L after 48 h adsorption by 10 mg L-CNSs, L-CNS activated by 5% NaOH exhibits a much higher SSA (214 m^2^/g) than the nonactivated L-CNS (26 m^2^/g), and the UV absorption intensity of the activated L-CNS is much lower. The enhancement of pollutant adsorption ability of L-CNSs by activation was due to the pores through Van der Waals interactions. Therefore, we conclude that under the high-temperature thermal treatment condition, alkali hydroxides can also act as a very useful chemical agent for carbon activation.

To be easily understood, rising carbonization temperature leads to a reduction in pore volume and an increase of the specific surface area [21]. The large specific surface area will result in the high adsorption ability of the annealed carbons. To achieve a high surface area, the lignin was preferred by annealing at high temperatures. However, it is also worth noting that the product yield is readily low at high carbonization temperatures. TsOH was found as an excellent pyrolysis catalyst to guarantee high carbon residual and well maintenance of the nanofibrous morphology during thermal decomposition of the nanofibrous carbon aerogels [8], which may also work for lignin carbonization. To verify this hypothesis, 5% TsOH was introduced into the lignin precursor to increase the product yield. As a result, with the same amount of freeze-dried lignin precursor, the L-CNS production prepared with 5% of TsOH at an annealing temperature of 1000 °C increases by 25.5% in comparison with the nonactivated L-CNS production (Table 3). In other words, TsOH promotes the pyrolysis instead of CO_2_ emission at low annealing temperature, thus, reducing the release of carbon resource. Moreover, little changes in specific surface area and RB adsorption capacity (Figures S5 and S6) were observed, that is, TsOH shows little effect on the properties and adsorption capacity of the L-CNS. This approach is also viable economically as it greatly increases the product yield. 

## 4. Conclusions

In conclusion, L-CNSs were directly prepared by freeze-drying and subsequent high-temperature annealing without activation operation, providing a novel and efficient biomass-derived adsorbent for pollutant removal in water treatment. It can be found that the higher specific surface area of the L-CNSs obtained by freeze-drying with more dilute lignin concentration and carbonization at high temperatures result in the better pollutant adsorption capacity. Moreover, the introduction of activating agents such as NaOH during the carbonization process significantly improves the SSA of obtained L-CNS and the resultant pollutant adsorption ability, acting a promising high porosity development. Besides, the pyrolysis catalyst such as TsOH helps to drastically enhance the product yield of the L-CNSs at the high-temperature carbonization. It is a promising approach to obtain sustainable low-cost adsorbents for pollution control. 

## Figures and Tables

**Table 1:** Properties of lignin and L-CNSs prepared under different conditions. Freeze-dried samples were prepared from 5 mg/mL lignin aqueous solutions.

Sample	Freezing Temp. (°C)	Carbonization Temp. (°C)	Specific Surface Area (m^2^/g)	Pore Volume (cm^3^/g)	RB Adsorption Capacity (mg/g) ^1^
lignin	– ^2^	– ^3^	0	0	– ^4^
L-C	– ^2^	1000	1	0.01	1.0
L-CNS@-20	–20	1000	204	0.09	32.9
L-CNS@-196	−196	1000	403	0.17	41.2

^1^ RB adsorption capacities of 2 mg L-CNSs in 10 mL of RB aqueous solution with initial RB concentration of 10 mg/L were determined after contact for 48 h. ^2^ Without FD. ^3^ Without carbonization. ^4^ Not measured.

**Table 2:** Properties of L-CNSs prepared by FD of lignin aqueous solutions, frozen at −196 °C, with varying lignin concentrations followed by carbonization at 1000 °C.

Sample	Concentration (mg/mL)	Surface Area (m^2^/g)	Pore Volume (cm^3^/g)	RB Adsorption Capacity ^1^ (mg/g)	Product Yield (%)
C-5	5	403	0.17	41.2	15.7
C-10	10	305	0.12	10.9	23.5
C-20	20	143	0.05	2.98	32.3
C-50	50	26	0.03	1.75	41.9

^1^ RB adsorption capacities of 2 mg of L-CNSs in 10 mL RB aqueous solution with initial RB concentration of 10 mg/L were determined after contact for 48 h.

**Table 3:** Properties of L-CNSs prepared by FD of 50 mg/mL lignin aqueous solutions, frozen at −196 °C, with different catalysts, followed by carbonization at 1000 °C.

Sample	Catalyst	Catalyst Content (%)	Lignin Concentration (mg/mL)	Surface Area (m^2^/g)	RB Adsorption Capacity ^1^ (mg/g)	Product Yield (%)
L-CNS	–	0	50	26	0.82	41.9
L-CNS/NaOH	NaOH	5	50	214	2.88	42.0
L-CNS/TsOH	TsOH	5	50	21	0.77	52.6

^1^ RB adsorption capacities of 10 mg L-CNSs in 10 mL RB aqueous solution with initial RB concentration of 10 mg/L were determined after contact for 48 h.

## Supplementary Materials

The following are available online at https://www.mdpi.com/2073-4360/12/4/797/s1, Figure S1: Images of lignin aqueous solution, respectively, prepared by frozen at −196 °C with a concentration of 2 mg/mL, at −20 °C with a concentration of 2 mg/mL, and at −20 °C with a concentration of 5 mg/mL. Figure S2: N_2_-adsorption isotherms of lignin-derived carbons, respectively, prepared by carbonization at 1000 °C with as-received alkali lignin, freeze-dried lignin frozen at −20 °C, and freeze-dried lignin frozen at −196°C. Figure S3: N_2_-adsorption isotherm of the lignin-derived carbons prepared by freeze drying of lignin aqueous solutions, frozen at −196 °C with varying concentrations of 50, 20, 10, and 5 mg/mL, followed by carbonization at 1000 °C. Figure S4: UV–Vis absorption spectra of RB solutions (initial RB conc. = 10 mg/L) before and after 48 h adsorption by 2 mg L-CNS. The absorbents were prepared by carbonization at 1000 °C without prior FD and with prior FD of solutions frozen at −196 °C. The concentrations of the freeze-dried solutions were 50, 20, 10, and 5 mg/mL. Figure S5: N_2_-adsorption isotherms behaviors of the lignin-derived carbon adsorbents prepared by freeze-drying of 50 mg/mL lignin aqueous solutions and subsequent different catalysts assistant annealing at 1000 °C. Figure S6: UV–Vis absorption spectra of RB solutions (initial RB conc. = 10 mg/L) before and after 48 h adsorption by 10 mg L-CNSs. The absorbents were prepared by FD of 50 mg/mL lignin aqueous solution, frozen at −196 °C, followed by carbonization at 1000 °C, without catalyst or catalyzed by NaOH and TsOH.
