# Supplementary material for: High-Yield Production of Lignin-Derived Functional Carbon Nanosheet for Dye Adsorption
Source: Polymers (Basel). 2020 Apr 2;12(4):797. doi: 10.3390/polym12040797 (PMC7240725; doi:10.3390/polym12040797)
Supplement: Supplementary file 1 [file polymers-12-00797-s001.docx]

Supplementary Materials for

**High-Yield Production of Lignin-Derived Functional Carbon Nano-sheet for Dye Adsorption**

*Fenggui Chen ^a,#^, Xi Hu ^a,#^, Xiaohan Tu ^a^, Linfei Chen ^a^, Linli Tan ^a^, Xi Liu ^a^, Yulin Mao ^a^, Jianwei Shi ^a^, Xiaoxu Teng ^a,^*, Shuhua He ^a^, Zonghui Qin ^a^, Jianhua Xu ^a^, Jian Wu ^b,^**

*^a^* *Chongqing Key Laboratory of Inorganic Special Functional Materials, School of Chemistry and Chemical Engineering, Yangtze Normal University, Fuling, Chongqing 408100, China*

*^b^* *Key Laboratory of Magnetic Materials and Devices, Ningbo Institute of Materials Technology and Engineering, Chinese Academy of Sciences, Ningbo 315201, China*

^#^ F. Chen and X. Hu contributed equally to this work.

*Correspondence authors: X.X. Teng (Email: tengxiaoxu@sina.com),

J. Wu (E-mail: jwu@nimte.ac.cn)


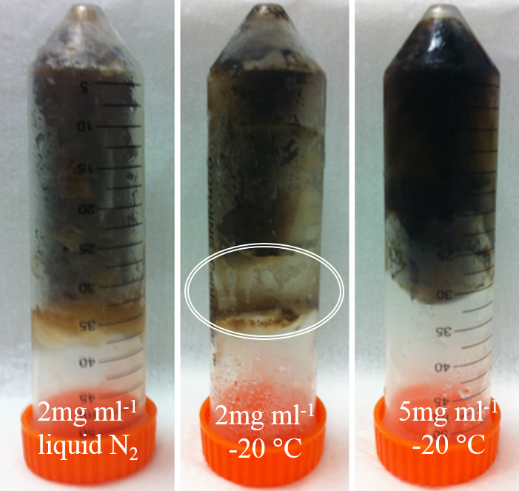


**Figure S1.** Images of lignin aqueous solution respectively prepared by frozen at -196 °C with a concentration of 2 mg/mL, at -20°C with a concentration of 2 mg/mL and at -20°C with a concentration of 5 mg/mL.

**

**

**Figure S2.** N_2_-adsorption isotherms of lignin-derived carbons respectively prepared by carbonization at 1000°C with as-received alkali lignin, freeze-dried lignin frozen at -20°C and freeze-dried lignin frozen at -196°C.


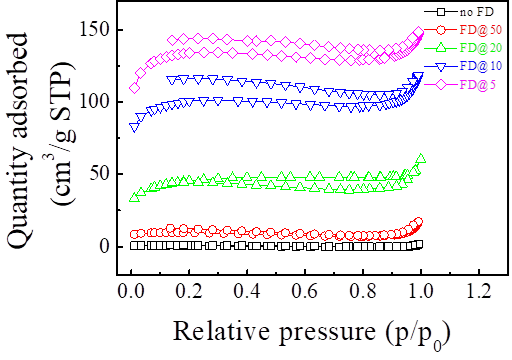


**Figure S3**. N_2_-adsorption isotherm of the lignin-derived carbons prepared by freeze-drying of lignin aqueous solutions, frozen at -196 °C with varying concentrations of 50, 20, 10, and 5 mg/mL, followed by carbonization at 1000°C.





**Figure S4.** UV-Vis absorption spectra of RB solutions (initial RB conc. = 10 mg/L) before and after 48 h adsorption by 2 mg L-CNS. The absorbents were prepared by carbonization at 1000°C without prior FD (FD) and with prior FD of solutions frozen at -196°C. The concentrations of the freeze-dried solutions were 50, 20, 10, and 5 mg/mL.


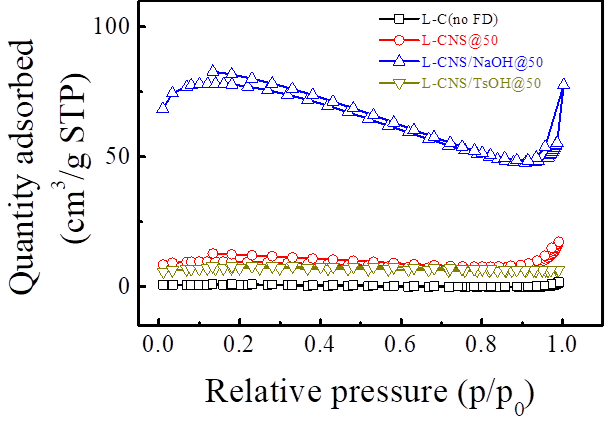


**Figure S5.** N_2_-adsorption isotherms behaviors of the lignin-derived carbon adsorbents prepared by freeze-drying of 50 mg/mL lignin aqueous solutions and subsequent different catalyst assistant annealing at 1000 °C.





**Figure S6.**  UV-Vis absorption spectra of RB solutions (initial RB conc. = 10 mg/L) before and after 48 h adsorption by 10 mg L-CNSs. The absorbents were prepared by FD of 50 mg/mL lignin aqueous solution, frozen at -196°C, followed by carbonization at 1000°C, without catalyst or catalyzed by NaOH and TsOH.
